# Using a Diverse Test Suite to Assess Large Language Models on Fast Health Care Interoperability Resources Knowledge: Comparative Analysis

**DOI:** 10.2196/73540

**Published:** 2025-08-12

**Authors:** Ahmad Idrissi-Yaghir, Kamyar Arzideh, Henning Schäfer, Bahadir Eryilmaz, Mikel Bahn, Yutong Wen, Katarzyna Borys, Eva Hartmann, Cynthia Schmidt, Obioma Pelka, Johannes Haubold, Christoph M Friedrich, Felix Nensa, René Hosch

**Affiliations:** 1 Institute of Diagnostic and Interventional Radiology and Neuroradiology, University Hospital Essen, Essen, Germany; 2 Institute for Artificial Intelligence in Medicine, University Hospital Essen, Hufelandstraße 55, Essen, 45147, Germany, 49 201 - 72377817; 3 Department of Central IT, Data Integration Center, University Hospital Essen, Essen, Germany; 4 Institute for Transfusion Medicine, University Hospital Essen, Essen, Germany; 5 Department of Pulmonary Medicine, University Hospital Essen - Ruhrlandklinik, Essen, Germany; 6 Department of Computer Science, University of Applied Sciences and Arts Dortmund, Dortmund, Germany; 7 Institute for Medical Informatics, Biometry, and Epidemiology, University Hospital Essen, Essen, Germany

**Keywords:** LLMs, medical informatics, health data interoperability, GPT-4, DeepSeek, large language models

## Abstract

**Background:**

Recent natural language processing breakthroughs, particularly with the emergence of large language models (LLMs), have demonstrated remarkable capabilities on general knowledge benchmarks. However, there is limited data on the performance and understanding of these models in relation to the Fast Healthcare Interoperability Resources (FHIR) standard. The complexity and specialized nature of FHIR present challenges for LLMs, which are typically trained on broad datasets and may have a limited understanding of the nuances required for domain-specific tasks. Improving health data interoperability can greatly benefit the use of clinical data and interaction with electronic health records.

**Objective:**

This study presents the Fast Healthcare Interoperability Resources (FHIR) Workbench, a comprehensive suite of datasets designed to evaluate the ability of LLMs to understand and apply the FHIR standard.

**Methods:**

In total, 4 evaluation datasets were created to assess the FHIR knowledge and capabilities of LLMs. These tasks include multiple-choice questions on general FHIR concepts and the FHIR Representational State Transfer (REST) application programming interface, as well as correctly identifying the resource type and generating FHIR resources from unstructured clinical patient notes. In addition, we evaluate open-source LLMs, such as Qwen 2.5 Coder and DeepSeek-V3, and commercial LLMs, including GPT-4o and Gemini 2, on these tasks in a zero-shot setting. To provide context for interpreting LLM performance, a subset of the datasets was human-evaluated by recruiting 6 participants with varying levels of FHIR expertise.

**Results:**

Our evaluation across multiple FHIR tasks revealed nuanced performance metrics. Commercial models demonstrated exceptional capabilities, with GPT-4o achieving a 0.9990 *F*_1_-score on the FHIR-ResourceID task, 0.9400 on the FHIR-QA task, and 0.9267 on the FHIR-RESTQA task. Open-source models also demonstrated strong performance, with DeepSeek-v3 achieving 0.9400 on FHIR-QA, 0.9400 on FHIR-RESTQA, and 0.9142 on FHIR-ResourceID. Qwen 2.5 Coder-7B-Instruct demonstrated high accuracy, scoring 0.9533 on FHIR-QA and 0.8920 on FHIR-ResourceID. However, all models struggled with the Note2FHIR task, with performance ranging from 0.0382 (OLMo) to a maximum of 0.3633 (GPT-4.5-preview), highlighting the significant challenge of converting unstructured clinical text into FHIR-compliant resources. Human participants achieved accuracy scores ranging from 0.50 to 1.0 across the first 3 tasks.

**Conclusions:**

This study highlights the competitive performance of both open-source models, such as Qwen and DeepSeek, and commercial models, such as GPT-4o and Gemini, in FHIR-related tasks. While open-source models are advancing rapidly, commercial models still have an advantage for specific, complex tasks. The FHIR Workbench offers a valuable platform for evaluating the capabilities of these models and promoting improvements in health data interoperability.

## Introduction

Large language models (LLMs) have revolutionized the field of natural language processing (NLP), showcasing unprecedented capabilities in understanding and generating human-like text across various domains [1-3]. Large models, such as GPT-4o [4] and LLaMA 3 [5] have demonstrated remarkable performance on general knowledge benchmarks, driving advances in applications ranging from conversational agents to automated content creation. In addition, these models have shown impressive results in medical tasks, where research has demonstrated the ability of GPT-4 to explain medical reasoning, tailor explanations for students, and generate counterfactual clinical scenarios [6]. Despite these successes, a notable challenge remains in evaluating and improving the performance of LLMs in specialized domains that require deep, structured knowledge representations [7]. This is particularly evident in the health care domain, where interoperability standards, such as the Fast Healthcare Interoperability Resources (FHIR), require the sophisticated integration of domain-specific data and a precise understanding of complex information structures.

FHIR is an interoperability standard developed by Health Level Seven International (HL7) for exchanging electronic health records and ensuring seamless interoperability between different health care systems [8]. Effective usage of FHIR is essential for improving clinical workflows, enabling precise data sharing, and driving innovation in health informatics. However, the complexity and specialized nature of FHIR present substantial challenges for LLMs, which are typically trained on broad datasets and may lack the nuanced understanding required for domain-specific tasks. In particular, a recent study [9] has shown that LLMs fine-tuned to clinical narratives can effectively transform unstructured clinical data into FHIR-compliant resources, highlighting their promising potential for improving interoperability in health care domain.

In order to advance research in this area, we present the FHIR Workbench, a comprehensive suite of datasets designed to assess and benchmark the knowledge and reasoning skills of LLMs concerning FHIR. The Workbench encompasses a range of tasks, including multiple-choice questions on general FHIR concepts and FHIR application programming interface (API) functionality, as well as resource recognition and FHIR resource generation from unstructured clinical notes. By providing a standardized evaluation framework, the FHIR Workbench aims to facilitate the systematic, future-proof evaluation and comparison of LLMs in the context of health care interoperability.

In addition, we are benchmarking and evaluating various LLMs, both open-source and commercial, in a zero-shot setting against the tasks provided by the FHIR Workbench. This evaluation aims to understand the current capabilities of these models in handling FHIR-specific tasks and to identify areas where improvements are needed.

By highlighting the state-of-the-art capabilities of LLMs in handling FHIR-specific tasks, this work aims to encourage further research and development in integrating advanced NLP techniques with health care standards. We envision that the FHIR Workbench will serve as a foundational tool for the community, driving progress toward more intelligent, reliable, and interoperable health care information systems.

## Methods

### Development of FHIR Task-Specific Datasets

#### Overview

To evaluate the performance of LLMs on FHIR-related tasks, we created and used 4 different datasets within the FHIR Workbench suite. Each dataset targets specific aspects of FHIR knowledge and application, ranging from basic conceptual understanding to practical resource generation from clinical notes. All datasets are publicly available and can be accessed on Hugging Face [10].

As 3 of the 4 datasets were fully or partially generated by artificial intelligence (AI), the questions and answers in our dataset were validated through a qualitative expert review process. Two FHIR experts with 5 and 6 years of experience in health care interoperability reviewed each question-answer pair for the FHIR-QA, FHIR-RESTQA, and Note2FHIR datasets. The experts evaluated technical accuracy, relevance to real-world FHIR implementation scenarios, and alignment with the FHIR specification. Disagreements were addressed through consensus discussions. To ensure dataset sufficiency, the questions were mapped to cover the core FHIR resources and interactions, with particular attention to areas commonly cited in FHIR implementation challenges. The dataset includes questions spanning structural, semantic, and operational aspects of the FHIR standard, covering the most common FHIR resources and interaction patterns.

#### FHIR-QA Dataset

The FHIR-QA dataset consists of 150 multiple-choice questions designed to assess general and fundamental knowledge of FHIR concepts. These questions cover a wide range of topics within the FHIR standard, from easy to challenging. Each question has 4 options with only 1 correct answer. The questions were generated using GPT-4o to cover different aspects of FHIR. To ensure accuracy and reliability, all questions and answers were validated and supported by FHIR experts. This dataset aims to test the general understanding of LLMs regarding FHIR principles, terminology, and basic functionality. The format of the questions follows a structured prompt template (see Textbox 1).

Textbox 1. **The prompt format for the multiple-choice tasks.**

**Prompt:**

Question: {{question}}

Options: A: {{mc_answer1}}

B: {{mc_answer2}}

C: {{mc_answer3}}

D: {{mc_answer4}} Answer:

#### FHIR-RESTQA Dataset

**Figure 1.**
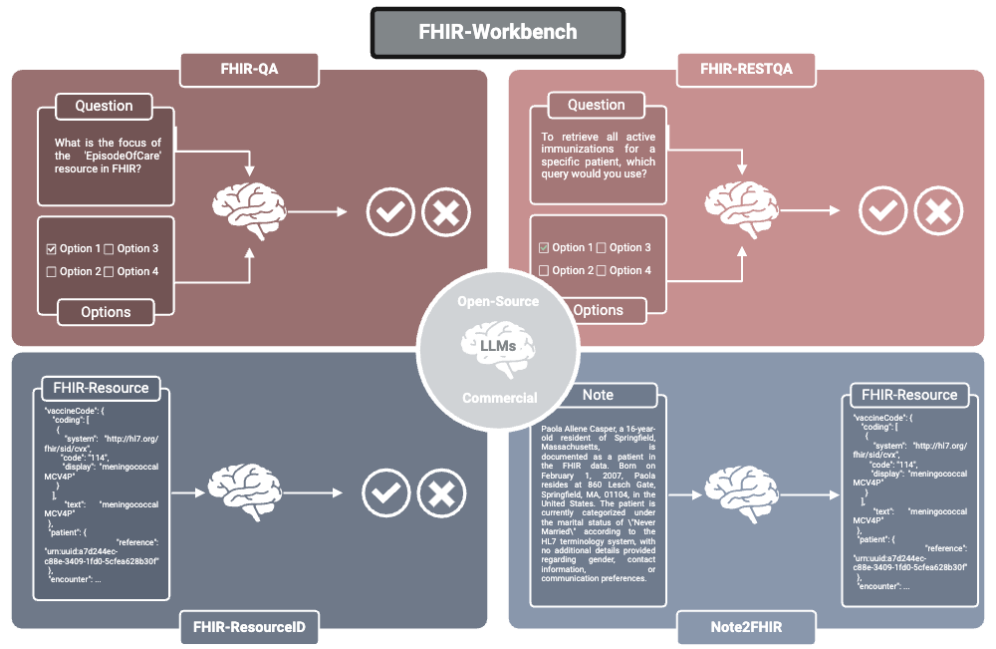
Exemplary visualization of all tasks contained in the Fast Healthcare Interoperability Resources workbench. Created in BioRender [11].

The FHIR-RESTQA dataset consists of 150 multiple-choice questions that require in-depth knowledge and understanding of the FHIR REST (Representational State Transfer) API and its structure. Designed to be more challenging than the FHIR-QA, these questions focus on advanced topics, such as API operations, data formats, and implementation details. The dataset was also generated using GPT-4o with prompts targeting the intricacies of the FHIR API. All questions and answers were reviewed and validated by FHIR experts to ensure accuracy and relevance. These questions are designed to assess the ability of LLMs to understand, analyze, and reason about the intricate functionalities and structures of the FHIR API (see Figure 1). This task follows the same prompt format as the FHIR-QA task.

#### FHIR-ResourceID Dataset

The FHIR-ResourceID dataset is designed to evaluate the ability of LLMs to identify and classify FHIR resources based on their JSON (JavaScript Object Notation) structure and content. The dataset contains 1000 examples derived from the “Oh Canada!” synthetic dataset available on SyntheticMass [12], which simulates Canadian patients spread across provinces. This synthetic dataset was generated using Synthea [13], a robust synthetic data generation framework for health care that simulates the lifespans of synthetic patients. Rather than creating new data, we repurposed existing synthetic records by applying a targeted preprocessing strategy tailored to LLM evaluation.

As a preprocessing step, the resourceType field was removed from each FHIR JSON resource, leaving the remaining structural and content-related elements intact. This transformation created a classification dataset where the input is the modified FHIR JSON, and the output label corresponds to the original FHIR resource type. The dataset spans 17 resource types, including Patient, Encounter, and others, providing a diverse and challenging set of examples. The questions were equally distributed across the 17 resource types. The dataset serves as a benchmark for assessing the ability of LLMs to recognize and differentiate FHIR resources based solely on their unique structural patterns and content in JSON format. The resource types are provided as options for the model to choose from. The question format follows the prompt template (see Textbox 2).

Textbox 2. **The prompt format for the resource identification task.**

**Prompt:**

{{resource}}

Question: What type of FHIR resource is this based on its structure?

Possible resource types (please choose exactly one):

Immunization, ImagingStudy, Patient, AllergyIntolerance, SupplyDelivery,

CareTeam, Procedure, DiagnosticReport, ExplanationOfBenefit, Condition,

Practitioner, Organization, Encounter, MedicationAdministration, Observation, MedicationRequest, CarePlan

Answer:

#### Note2FHIR Dataset

The Note2FHIR dataset consists of 150 synthetic samples designed to evaluate the ability of LLMs to generate structured FHIR resources from unstructured clinical text. To build the dataset, we reused the FHIR resources from the publicly available “test-fhir” dataset [14], which includes synthetic FHIR data along with associated clinical notes. However, we did not use the original notes from that dataset, as they were often provided as bullet points or fragments and were unsuitable for the task.

Instead, we generated new clinical notes from the FHIR JSONs using GPT-4o. These generated notes were then presented to the LLMs, which were prompted to reconstruct the original FHIR resources. The goal of this setup is to assess whether LLMs can correctly extract structured information and generate syntactically valid and semantically appropriate FHIR representations. The prompt format used for this task is shown in Textbox 3.

Textbox 3. **The prompt template for the Note2FHIR task.**

**Prompt:**

Given the following patient note, generate the corresponding FHIR (Fast Healthcare Interoperability Resources) JSON representation. Please ensure that:

- All keys and string values are enclosed in double quotes.
- The JSON is valid and follows proper syntax.
- There are no comments or code expressions.
  Enclose the JSON output within triple backticks with ‘json’ specified, like this ```json
  <YourJSON here>

```

Make sure the first resourceType is Bundle.

Patient Note:

{{note}}

The Note2FHIR task aims to challenge LLMs for effectively interpreting the clinical narratives from the notes and generate accurate, structured FHIR resource outputs in JSON format. Evaluating this capability is key to improving the interoperability of health care data systems by bridging the gap between unstructured text and structured data.

Since the clinical notes were generated by GPT-4o, the clinical and semantic accuracy of the notes cannot be guaranteed. The FHIR resources and notes were manually reviewed by FHIR experts, but since they only have experience in medical informatics and no classical medical training, the clinical accuracy of the notes could not be thoroughly verified.

The integration of these datasets into the FHIR Workbench creates a robust evaluation framework for LLMs within the FHIR domain. Addressing a range of challenges, from basic knowledge testing to complex data transformation tasks, each dataset provides unique insights into the strengths and limitations of different models. This comprehensive approach ensures a thorough evaluation of their ability to handle FHIR-related scenarios.

### Evaluation

This section outlines the evaluation methods and metrics used to assess the performance of the various LLMs on the tasks provided by FHIR Workbench. To ensure consistent and systematic benchmarking, the language model evaluation harness (LM Eval Harness) [15] was used as the evaluation framework. This framework enables uniform testing across various tasks and has been extended to include the new FHIR-specific tasks introduced by the FHIR Workbench. By integrating these tasks into the framework, reproducible and comparable results across different open-source LLMs were enabled, ensuring a robust and standardized evaluation process.

Appropriate evaluation metrics were selected for each task based on the nature of the task and the type of outputs expected.

For the multiple-choice question tasks, FHIR-QA and FHIR-RESTQA, accuracy was used as the primary evaluation metric. Accuracy was calculated as the percentage of questions for which the model selected the correct answer out of the total number of questions. The evaluation of the multiple-choice questions in the LM evaluation harness is performed by comparing the log-likelihood, which measures the probability of the given output string conditional on the given input, providing a deeper insight into the confidence of the model in its predictions.

In the FHIR-ResourceID task, the objective is to classify FHIR JSON resources into their corresponding resource types. The standard *F*_1_-score, computed using microaveraging, was used as the evaluation metric. This metric was chosen to comprehensively assess classification performance by accounting for both the correctness of predictions and the ability to identify all instances of each resource type.

The Note2FHIR task requires models to generate FHIR resources from unstructured clinical notes in JSON format. As a preliminary step, we apply preprocessing using regular expressions to extract valid JSON from the generated text and correct common formatting errors (eg, trailing commas). This step is necessary to enable downstream semantic analysis but does not influence the evaluation metrics. The core evaluation focuses on assessing the semantic correctness and completeness of the generated FHIR structures. Rather than relying on simple string-based matching, which would miss semantically equivalent but syntactically different representations, we use a semantic comparison approach. Both the predicted and reference FHIR resources are flattened into sets of dot-delimited key-value pairs (eg, “Patient.name.given” → “John,” “Observation.valueQuantity.value” → “120”). This flattening preserves the hierarchical structure and semantic relationships within the FHIR resources while enabling systematic comparison. We compute an *F*1-based similarity score that evaluates three aspects of semantic correctness: (1) Completeness—measuring how many clinically relevant elements from the reference are captured in the prediction (recall), (2) Precision—assessing whether the generated elements are appropriate and free from extraneous or incorrect clinical information, and (3) Structural Alignment—evaluating whether clinical concepts are mapped to the correct FHIR elements and hierarchical positions. This approach captures both the semantic content (what clinical information is represented) and structural validity (how it is organized within the FHIR framework) more accurately than direct string comparison, providing a comprehensive assessment of the models’ ability to generate clinically meaningful and structurally correct FHIR resources.

Applying these evaluation metrics within the LM evaluation harness ensures a consistent and robust evaluation of LLM performance on FHIR Workbench tasks. This approach facilitates the identification of strengths and weaknesses across different models, paving the way for targeted improvements in LLM capabilities within the health care interoperability domain.

### LLM Selection

LLMs were selected to cover multiple dimensions relevant to the evaluation of FHIR tasks. We included models with different parameter scales (7B to 671B) to evaluate how model size affects performance on domain-specific tasks, from efficient smaller models suitable for local use to large models with extensive capabilities. The selection includes both general models (Llama-3.1, GPT-4o, and Gemini) and domain-specific variants (BioMistral for biomedical tasks and Qwen 2.5 Coder for structured data) to evaluate whether domain-specific pretraining offers advantages for FHIR-related tasks. We included some of the major model families based on established benchmarks and community acceptance to ensure that different architectural approaches are covered, including dense models and Mixture-of-Experts (MoE) architectures (DeepSeek-v3). Both open-source models and proprietary systems were included to gain insight into different deployment scenarios and establish performance baselines across the accessibility spectrum. This broad selection enables the analysis of the relationship between model characteristics (size, specialization, and architecture) and performance in health informatics tasks. In Table 1, a list of all evaluated models is provided.

**Table 1.** Large language models selected for the experiments.

LLM^a^	Size	License	Open vs Closed
Llama-3.1	8B	Llama 3.1 Community License Agreement	Open Weights
Qwen 2.5 Coder	7B/32B	Apache 2.0	Open Weights
Mistral Small 3	24B	Apache 2.0	Open Weights
Gemma 2	9B	Gemma	Open Weights
BioMistral	7B	Apache 2.0	Open Weights
OLMo	7B	Apache 2.0	Open Weights
Tülu3	8B	Llama 3.1 Community License Agreement	Open Weights
Phi-4	14B	MIT License (Massachusetts Institute of Technology License)	Open Weights
DeepSeek-v3	671B (Mixture of Experts)	Apache 2.0	Open Weights
GPT-4o / GPT-4.5	Unspecified	Proprietary	Closed
Gemini-1.5/2	Unspecified	Proprietary	Closed

a LLM: large language model.

Among the open-source models evaluated, LLaMA 3.1 [5] stands out as an advanced iteration of the LLaMA series, known for its efficiency and scalability in handling large-scale language tasks. Mistral Small 3 represents the third iteration of the Mistral [16] model, offering robust instruction-following and language generation capabilities at a fraction of the size and latency of larger models, all while remaining highly efficient for a broad spectrum of generative AI tasks. Qwen 2.5 Coder [17] is a series of code-specific language models developed by Alibaba Cloud’s Qwen team. Building upon the Qwen 2.5 [18] architecture, these models offer a versatile balance between performance and computational requirements, providing flexibility across various application scenarios. Gemma [19] is a family of lightweight, state-of-the-art open models developed by Google DeepMind. Built upon the same research and technology as the Gemini models, Gemma excels in language understanding, reasoning, and safety. BioMistral [20] is an open-source LLM tailored specifically for the biomedical domain. Building upon the Mistral foundation model, it underwent further pretraining on PubMed Central data to enhance its proficiency in understanding and generating domain-specific content. OLMo [21] is a fully open, competitive language model that provides its entire training data, along with training and evaluation code, for unrestricted research. By removing the barriers of proprietary models, it enables rigorous studies of biases and potential risks, fueling the next wave of NLP innovation. Tülu 3 [22] is an open-source, instruction-following language model series developed by the Allen Institute for AI. Building on the Llama 3.1 base models, it uses advanced posttraining techniques such as supervised fine-tuning and direct preference optimization [23] to improve capabilities in the areas of instruction-following, reasoning, mathematics, coding, and security. Phi-4 [24] is a 14-billion-parameter language model that focuses on data quality. Unlike many models that rely primarily on organic data such as web text or code, phi-4 strategically integrates synthetic data throughout its training. DeepSeek-V3 [25] is a cutting-edge MoE language model featuring 671 billion total parameters, with 37 billion activated per token. It adopts Multi-head Latent Attention and DeepSeek MoE architectures to achieve efficient inference and cost-effective training. For the commercial LLMs, we evaluated GPT-4o [4], the preview of GPT-4.5 [26] and Gemini [27]. GPT-4o, developed by OpenAI, is well-known for its advanced language understanding and generation capabilities, demonstrating versatility across a wide range of tasks, including complex reasoning and contextual comprehension. GPT-4.5 is OpenAI’s most extensive and versatile model yet, building on GPT-4o while scaling pretraining further. It uses new supervision techniques alongside familiar methods (supervised fine-tuning and reinforcement learning from human feedback) to broaden its knowledge base and improve alignment with user intent. Gemini is a cutting-edge model developed by Google DeepMind known for its high accuracy and efficiency in processing specialized tasks, positioning itself as a strong competitor in the commercial LLM landscape.

All selected models were evaluated in a zero-shot setting on the FHIR Workbench tasks, using the instruction-tuned or chat-optimized versions of the base models rather than the base models themselves, without any additional fine-tuning specific to the FHIR standard. This approach ensures that the scores reflect the inherent capabilities and generalizability of the models. For all tasks, default hyperparameters were used, except for the Note2FHIR task, where the maximum token limit was set to 8192 to maintain consistency across models, as they do not all support the same maximum token generation.

### Human Validation

To provide context for interpreting LLM performance and compare the results to a human baseline in FHIR-related tasks, we conducted human evaluation across our benchmark datasets. We recruited 6 human evaluators with varying levels of FHIR expertise and organized them into three groups to assess performance across different experience levels.

Group 1 consisted of 2 FHIR experts with 7 and 6 years of experience in medical informatics and FHIR implementation. Group 2 included 2 moderately experienced practitioners with 3 and 1 year of FHIR developer experience. Group 3 comprised 2 data scientists with 3 and 1 year of experience in conducting AI research using FHIR data, representing domain users with technical but less specialized knowledge of FHIR. The participants had no prior contact with the datasets and were not involved in any quality control steps.

Each group evaluated 50 questions from three datasets: FHIR-QA, FHIR-RESTQA, and FHIR-ResourceID. The datasets were divided into 3 equally sized segments, with each group assigned 1 segment per dataset to prevent overlap and maintain evaluation independence. For FHIR-QA and FHIR-RESTQA, which contain 150 questions each, the complete datasets were evaluated across all 3 groups. For the FHIR-ResourceID, which contains a total of 1000 questions, we randomly selected 150 questions (50 per group) while maintaining diversity and balance across FHIR resource types by proportionally sampling from each resource category. A web-based application was developed for this purpose, providing an interface for users to answer questions. In addition, to assess the difficulty of the questions, participants had to rate how difficult each question was to answer. This further gives information about the overall difficulty and quality of the questions. Participants were instructed to rely solely on their existing knowledge without consulting external resources, internet sources, or collaborating with others.

### Ethical Considerations

This study was approved by the Ethics Committee of the Medical Faculty of the University of Duisburg-Essen (approval number 23‐11557-BO). Due to the study’s retrospective nature, the requirement of written informed consent was waived by the Ethics Committee of the Medical Faculty of the University of Duisburg-Essen. All methods were carried out in accordance with relevant guidelines and regulations.

## Results

### LLM Performance

This section presents the findings from benchmarking and evaluating the selected LLMs using the FHIR Workbench suite. The results are organized according to the different tasks within the benchmark, providing a comprehensive overview of each model’s performance across various aspects of FHIR knowledge and application (see Table 2). Accuracy is used as the evaluation metric for the QA tasks, while the resource identification task is assessed using the micro *F*_1_-score. The Note2FHIR task is evaluated based on a dictionary-based *F*_1_-score for similarity. Due to the high computational cost of GPT-4.5, its performance on the FHIR-ResourceID task was not evaluated.

**Figure 2.**
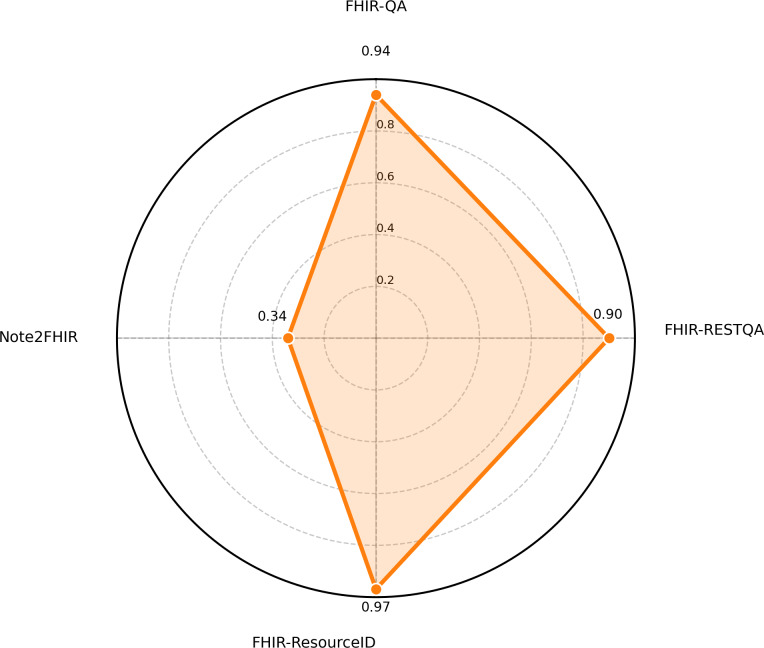
Radar chart of the performance of Gemini-2-Flash on FHIR (Fast Healthcare Interoperability Resources) workbench tasks.

In our evaluation, each model was scored on 4 tasks, FHIR-QA, FHIR-RESTQA, FHIR-ResourceID, and Note2FHIR, to assess how accurately and consistently it performed on FHIR-related tasks. Overall, the results show that the performance of the LLMs tested varied considerably, with some models performing well on some metrics but struggling on others. The radar charts (Figures2  3) compare the performance of the top 2 models across the 4 key FHIR-related tasks: FHIR-QA, FHIR-RESTQA, FHIR-ResourceID, and Note2FHIR. Figure 4 illustrates the performance comparison among the 5 top-performing LLMs on various tasks. It highlights the strengths and weaknesses of each model, showcasing variations in their ability to handle different aspects of FHIR-based tasks.

A clear pattern from Table 2 is the consistently high scores (above 0.90) on the FHIR-QA, FHIR-RESTQA, and FHIR-ResourceID tasks among many proprietary models, such as Gemini-1.5-Pro and Gemini-1.5-Flash. Surprisingly, however, GPT-4.5-preview trails its earlier GPT-4o variants in the Questions and answers tasks, an unexpected drop given its more advanced iteration. Among the open-source LLMs, Qwen 2.5 Coder and DeepSeek-v3 show strong, balanced performance across FHIR-QA, FHIR-RESTQA, and FHIR-ResourceID—especially DeepSeek-v3, which exceeds 0.94 in several tasks. In contrast, some open-source models (such as BioMistral and OLMo) struggle with FHIR-ResourceID, although they perform well on FHIR-QA and FHIR-RESTQA. Meanwhile, Gemma 2 performs well on FHIR-RESTQA and excels on FHIR-ResourceID but surprisingly underperforms on FHIR-QA with an accuracy of only 0.5733.

**Table 2.** Performance comparison of various large language models on different Fast Healthcare Interoperability Resources Workbench tasks in a zero-shot setting.

LLM^a^	FHIR-QA (Acc^b^)	FHIR-RESTQA (Acc)	FHIR-ResourceID (*F*_1_-score)	Note2FHIR (*F*_1_-score)
meta-llama/Llama-3.1-8B-Instruct	0.8533	0.8800	0.8200	0.2078
Qwen/Qwen2.5-Coder-7B-Instruct	0.9533^c^	0.8733	0.8920	0.2121
mistralai/Mistral-Small-24B-Instruct-2501	0.8866	0.9200	0.8860	0.3401
google/gemma-2-9b-it	0.5733	0.8200	0.9520	0.0629
BioMistral/BioMistral-7B-DARE	0.8533	0.8400	0.5810	0.0764
allenai/OLMo-2-1124-7B-Instruct	0.8200	0.7400	0.6110	0.0382
Qwen/Qwen2.5-Coder-32B-Instruct-AWQ	0.9000	0.9133	0.8880	0.3354
allenai/Llama-3.1-Tulu-3-8B	0.8333	0.8533	0.7320	0.2029
microsoft/phi-4	0.8866	0.8933	0.8290	0.2899
DeepSeek-v3	0.9400	0.9400^c^	0.9142	0.3223
Gemini-1.5-Flash	0.9200	0.9067	0.9200	0.2415
Gemini-1.5-Pro	0.9333	0.9133	0.9370	0.3431^c^
Gemini-2-Flash	0.9400	0.9000	0.9690^c^	0.3400
GPT-4o-mini^d^	0.9533	0.9400	0.9208	0.1634
GPT-4o1	0.9400	0.9267	0.9990	0.3474
GPT-4.5-preview1	0.9067	0.9200	—^e^	0.3633

a LLM: large language model.

b Acc: Accuracy.

c Best scores.

d It is important to acknowledge that GPT-4o was used to generate the FHIR-QA, FHIR-RESTQA, and Note2FHIR datasets in this evaluation. This introduces a potential source of bias because GPT-4o may have an inherent advantage when evaluated on content created using the same model architecture. This could manifest as familiarity with specific patterns, terminology, or reasoning approaches present in the test examples.

e Not available.

**Figure 3.**
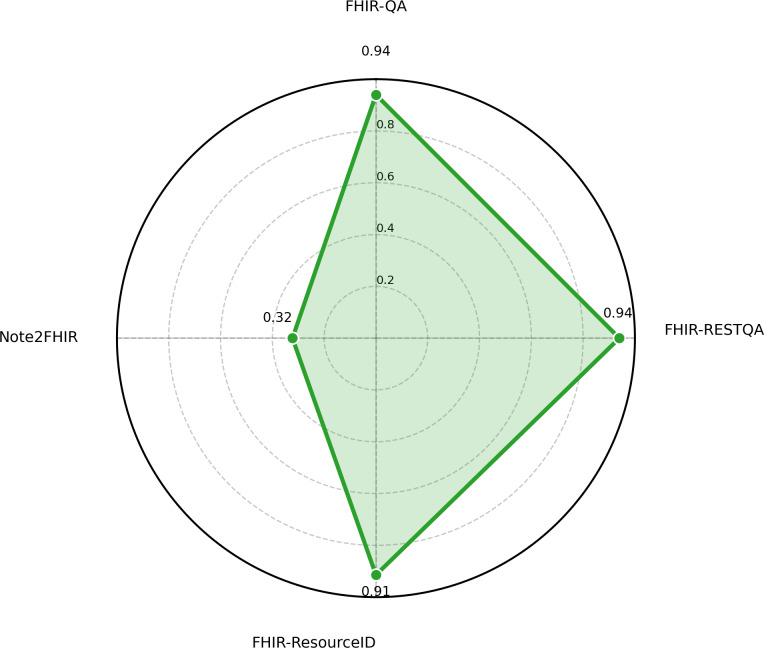
Radar chart of the performance of DeepSeek-v3 on FHIR (Fast Healthcare Interoperability Resources) workbench tasks.

**Figure 4.**
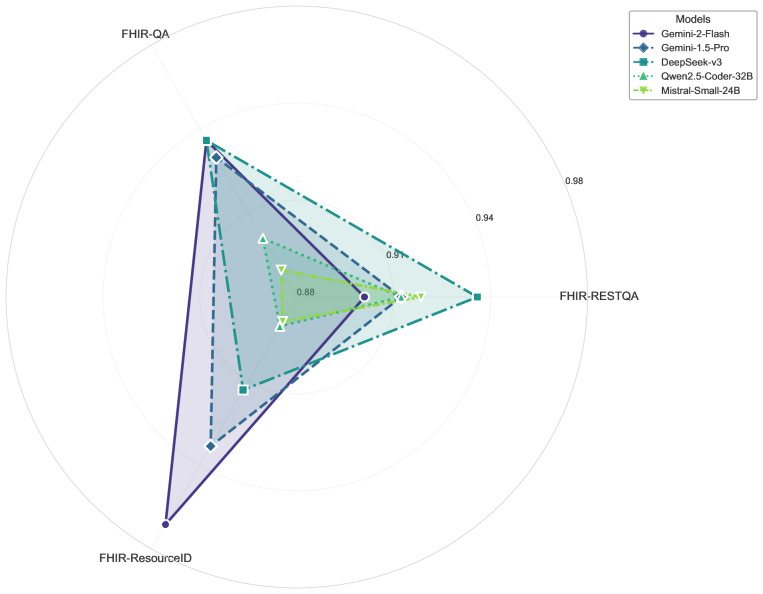
Comparison of the top five performing large language models on FHIR (Fast Healthcare Interoperability Resources) workbench tasks.

Performance on Note2FHIR remains consistently lower across the board, even for models that excel in other areas. For example, GPT-4o scores 0.3474, while GPT-4.5-preview leads with 0.3633 but still leaves room for improvement in mapping unstructured clinical notes to FHIR. Among the open-source options, Qwen 2.5 Coder-32B and DeepSeek-v3 score 0.3354 and 0.3223, respectively, indicating comparable results to their proprietary counterparts. Meanwhile, others, such as OLMo, score notably low (0.0382), highlighting the overall complexity of Note2FHIR. Among the proprietary models excluding the GPT‑4 series, the best result comes from Gemini‑1.5‑Pro (0.343), narrowly ahead of Gemini‑2‑Flash (0.340). The strongest open‑source contender, Mistral‑Small‑24B, matches Gemini‑2‑Flash at 0.340, while Qwen 2.5 Coder‑32B (0.335) and DeepSeek‑v3 (0.322) trail by only 0.01-0.02 points.

It is important to acknowledge that GPT-4o was used to generate the FHIR-QA, FHIR-RESTQA, and Note2FHIR datasets in this evaluation. This introduces a potential source of bias because GPT-4o may have an inherent advantage when evaluated on content created using the same model architecture. This could manifest as familiarity with specific patterns, terminology, or reasoning approaches present in the test examples.

### Human Results

In this section, the results of human participants are presented. The results are listed for each task and participant. Additional information about the background and experience of each participant is also provided. The results of the participants for the 3 FHIR benchmark tasks are displayed in Table 3. All accuracy scores were calculated by dividing the number of correctly answered questions by the total number of questions.

**Table 3.** Performance comparison of various Fast Healthcare Interoperability Resources experts and data scientists on the different datasets.

Participant number	Group	Background/Experience	FHIR^a^-QA (Acc)^b^	FHIR-RESTQA (Acc)	FHIR-ResourceID (Acc)
Participant 1	Group 1	FHIR Developer (6 y experience)	0.80	1.0	0.86
Participant 2	Group 1	FHIR Developer (7 y experience)	0.94	0.88	0.94
Participant 3	Group 2	FHIR Developer (3 y experience)	0.88	0.82	0.68
Participant 4	Group 2	FHIR Developer (1 y experience)	0.78	0.56	0.50
Participant 5	Group 3	Data Scientist (3 y experience)	0.86	0.90	0.88
Participant 6	Group 3	Data Scientist (1 y experience)	0.76	0.60	0.50

a FHIR: Fast Healthcare Interoperability Resources.

b Acc: Accuracy.

The participants achieved accuracy scores ranging from 0.76 to 0.94 for the FHIR-QA dataset. The best score, 0.94, was achieved by participant 2, a FHIR developer with 7 years of experience in the field. The same accuracy score could be reached by the same participant for the FHIR-ResourceID task. For the FHIR-RESTQA dataset, the best score was achieved by participant 1, a 6-year experienced FHIR developer, who reached an accuracy score of 1.0.

The worst scores were obtained for the FHIR-QA (0.76) and FHIR-ResourceID (0.50) tasks by participant 6, who had a 1-year background in data science. The FHIR-RESTQA dataset was answered worse by participant 4, who also has 1 year of FHIR developer experience.

In addition to these scores for the human participants, they were also required to rate the difficulty of each question. The results are listed in Table S1 in Multimedia Appendix 1. For further information about the quality of the datasets, the number and percentage of overlapping answers are presented in Table 4. Agreement was measured by counting the number of matching answers given by each participant within a group. Correct overlap refers to the number of matching answers that were answered correctly, while wrong overlap refers to the number of matching answers that were answered incorrectly.

**Table 4.** Overlapping answers of each group.

Evaluation Task		Group 1 (Total=50)	Group 2 (Total=50)	Group 3 (Total=50)
FHIR^a^-QA, n (%)				
Total overlap		41 (82)	40 (80)	38 (76)
Correct overlap		39 (78)	37 (74)	36 (72)
Wrong overlap		2 (4)	3 (6)	2 (4)
FHIR-RESTQA, n (%)				
Total overlap		44 (88)	25 (50)	29 (58)
Correct overlap		44 (88)	23 (46)	28 (56)
Wrong overlap		0 (0)	2 (4)	1 (2)
FHIR-ResourceID, n (%)				
Total overlap		46 (92)	23 (46)	22 (44)
Correct overlap		43 (86)	20 (40)	22 (44)
Wrong overlap		3 (6)	3 (6)	0 (0)

a FHIR: Fast Healthcare Interoperability Resources.

With a total overlap of 82% (41/50) for the FHIR-QA, 88% (44/50) for the FHIR-RESTQA, and 92% (46/50) for the FHIR-ResourceID tasks, group 1 had the highest agreement on answers. Of these overlapping answers, 78% (39/50; FHIR-QA), 88% (44/50; FHIR-RESTQA), and 86% (43/50; FHIR-ResourceID) were correct.

The overall agreement of answers was lower for other groups, especially for the FHIR-RESTQA and FHIR-ResourceID tasks, with a total overlap of 50% and 46% (23/50) for group 2% and 58% and 44% (22/50) for group 3.

## Discussion

### Principal Findings

Overall, the results show that LLMs perform well on tasks requiring general FHIR knowledge, such as those assessed in the FHIR-QA and FHIR-RESTQA datasets. Models, such as Qwen 2.5 Coder and DeepSeek-v3, consistently achieve high accuracy in multiple-choice questions, illustrating their robust ability to understand and reason about basic FHIR concepts and API functionality. GPT-4.5-preview also performs strongly in these zero-shot settings; however, it surprisingly falls slightly behind its predecessor on the question-and-answer tasks, a noteworthy result given its more advanced iteration. These high scores underscore how modern LLM architectures can generalize even in specialized domains.

Despite these strengths, the results reveal notable limitations when deeper reasoning, structured generation, or domain-specific alignment is required. The Note2FHIR task, for instance, sees much lower performance across all models, including GPT-4.5-preview and GPT-4o, underscoring the challenges of converting unstructured clinical text into accurate FHIR resources. Even high-performing systems struggle to maintain structural and semantic fidelity, underscoring the difficulty of integrating clinical context and adhering to standardized schemas. Furthermore, the significant performance disparities in FHIR-ResourceID for specific models, such as Biomistral, suggest that some LLMs lack the ability to recognize subtle JSON-based structural nuances, likely due to limited domain-specific training in zero-shot conditions.

These results also highlight the performance differences between open-source and commercial models. While proprietary models, such as GPT-4o and Gemini consistently perform well, open-source systems, such as DeepSeek-v3 and Qwen 2.5 Coder, remain highly competitive, with DeepSeek-v3 outperforming commercial alternatives on several tasks. This highlights how factors like training data quality, computational resources, and architectural choices can be more decisive than whether a model is open-source or proprietary.

The human evaluation provides valuable context for interpreting LLM performance, with participants achieving accuracy scores ranging from 0.50 to 1.0 across datasets. Performance was clearly correlated with FHIR expertise, as the most experienced developers (Group 1) consistently outperformed less specialized participants, achieving individual scores of up to 0.94‐1.0. Data scientists showed competitive performance on some tasks but struggled notably with FHIR-ResourceID, suggesting this task requires more specialized domain knowledge.

The high agreement rates among experienced practitioners (82%‐92% overlap with 78%‐88% correct agreements) give potential insights into the dataset quality. Lower agreement in less experienced groups reflects the inherent difficulty of FHIR tasks without deep domain expertise. Comparisons between human and LLM performances are difficult because human participants only answered a subset of questions each. In addition, statements about human performance are not generalizable because the participants all had different levels of FHIR expertise and experience.

Looking ahead, improving LLM capabilities for health care interoperability will require several strategies. First, low scores on Note2FHIR suggest a need for domain-specific fine-tuning or hybrid approaches that combine LLMs with symbolic or rule-based methods to ensure accurate structured output. Specialized pretraining on clinical text and FHIR resources could also enhance the models’ ability to generate health care–specific content. Second, architectural innovations that directly handle structured data, such as schema-aware attention or integration with graph neural networks, may improve accuracy when working with complex JSON-based FHIR tasks. Incorporating external knowledge bases (eg, SNOMED CT [Systematized Nomenclature of Medicine–Clinical Terms] or *ICD-10* [*International Statistical Classification of Diseases, Tenth Revision*]) could boost the contextual understanding of clinical information. Finally, the FHIR Workbench offers a robust and reproducible framework for tracking progress in health care–oriented LLMs. Future expansions might incorporate additional tasks, such as FHIR resource validation or the seamless integration of LLMs with clinical systems, further driving advancements in health care interoperability.

### Limitations

This study has certain limitations that should be considered when interpreting the results. All evaluations were conducted under zero-shot conditions, which may not fully capture the potential of models that benefit from domain adaptation or fine-tuning. Excluding fine-tuning could mean some models performed below their optimal capability for real-world applications.

In addition, many of the open-source models examined here were relatively small in scale (generally under 20 billion parameters), which may have contributed to their lower performance compared to larger commercial counterparts. Model size and training resources are particularly influential in specialized domains such as health care interoperability.

The synthetic nature of the test datasets, while controlled and reproducible, may not accurately reflect the variability and complexity of real-world clinical documentation. Expanding the evaluation to include a broader range of real-world clinical narratives and additional resource types would provide a more comprehensive assessment of model performance in practical settings. In particular, the Note2FHIR dataset, which contains synthetically generated clinical notes, may not be as accurate and correct as real-world documents. The future use of FHIR resources and real-world clinical documents may lead to more representative results for this task. Our evaluation approach focused primarily on technical correctness rather than clinical accuracy. We assessed the structural validity of the generated FHIR resources through automated JSON parsing and *F*_1_ similarity scoring against reference resources. While this methodology effectively evaluates syntactic correctness and structural adherence to the FHIR standard, it does not fully capture potential clinically significant errors or semantic misrepresentations that might occur in the generated resources. The preprocessing step to ensure valid JSON format was necessary for evaluation, but we acknowledge that in real-world applications, the ability to generate semantically correct and complete FHIR structures is equally important. Our evaluation metrics do not specifically differentiate between minor syntactic errors and more significant semantic or clinical errors that could impact patient care. A more complete semantic evaluation could strengthen the evaluation framework. This step must be performed manually by humans, as all evaluated LLMs demonstrated a general understanding of the FHIR standard but lacked the ability to adapt it for medical documentation.

Finally, a large portion of the evaluation datasets (FHIR-QA, FHIR-RESTQA, and Note2FHIR) were generated using GPT-4o, which introduces the potential for bias favoring models from the GPT-4 family. This bias can manifest as patterns in language use, argumentation approaches, or area-specific representations in the datasets. However, GPT-4o did not always receive the highest scores on all evaluation tasks. Models, such as DeepSeek-v3 or Gemini-1.5-Pro, demonstrated competitive and even superior performance in some domains. These results suggest that these models may be capable of performing well despite potential biases in the generation process. The extent and impact of any bias introduced through GPT-4o dataset generation remain an open question that warrants further investigation. Future research should consider alternative dataset generation methodologies involving multiple diverse models or human experts to minimize potential sources of bias when evaluating different model architectures and training paradigms.

### Conclusions

In this study, we present FHIR-Workbench, a comprehensive suite of datasets designed to benchmark LLMs against FHIR. The tasks range from general and API-specific multiple-choice questions to resource recognition and clinical note-based generation, providing a diverse platform for testing how effectively LLMs comprehend and apply complex FHIR standards. Our findings indicate that commercial and large open-source models achieve comparable performances. Smaller open-source models, in general, achieved slightly lower scores, especially those not fine-tuned on domain-specific data.

All FHIR-Workbench code is publicly available on GitHub [28], encouraging further research and development in health care interoperability. Future work will expand FHIR-Workbench to include real-world clinical data, additional resource types, and fine-tuning strategies to enhance the performance of open-source models. Through these efforts, we aim to enhance the integration of NLP within health care systems, thereby fostering more effective and standardized clinical data management.

## Supplementary material

10.2196/73540 Multimedia Appendix 1 Human participant ratings of question difficulty.

## Abbreviations

AI: artificial intelligence
API: application programming interface
FHIR: Fast Healthcare Interoperability Resources
HL7: Health Level Seven International
*ICD-10*: *International Statistical Classification of Diseases, Tenth Revision*
LLM: large language model
MoE: Mixture-of-Experts
NLP: natural language processing
REST: Representational State Transfer
SNOMED CT: Systematized Nomenclature of Medicine–Clinical Terms
