# Supplementary material for: Using a Diverse Test Suite to Assess Large Language Models on Fast Health Care Interoperability Resources Knowledge: Comparative Analysis
Source: J Med Internet Res. 2025 Aug 12;27:e73540. doi: 10.2196/73540 (PMC12360669; doi:10.2196/73540)
Supplement: Multimedia Appendix 1 [file jmir-v27-e73540-s001.docx]

Supplementary Information

# Supplementary Tables

**Supplementary Table 1**. Difficulty ratings of human participants. The participants should rate the difficulty of every question in the human evaluated dataset. They had the option for three difficulty levels: easy, medium or hard. The participants all gave varying difficulty ratings. While participant 1 rated 45 questions as easy for the FHIR-QA dataset, participant 6 found only nine questions easy and 28 hard. In general, the FHIR-RESTQA was considered to be the most difficult dataset with 19.67 average easy ratings. However, the most hard ratings with an average of 12,67 were given to the FHIR-ResourceID task.

|  | FHIR-QA | | | FHIR-RESTQA | | | FHIR-ResourceID | | |
| --- | --- | --- | --- | --- | --- | --- | --- | --- | --- |
|  | Easy | Me-  dium | Hard | Easy | Me-  dium | Hard | Easy | Me-  dium | Hard |
| Participant 1 | 45 | 4 | 1 | 21 | 21 | 8 | 50 | 0 | 0 |
| Participant 2 | 21 | 25 | 4 | 25 | 21 | 4 | 20 | 25 | 5 |
| Participant 3 | 32 | 13 | 5 | 21 | 26 | 3 | 17 | 22 | 11 |
| Participant 4 | 11 | 18 | 21 | 15 | 28 | 7 | 16 | 18 | 16 |
| Participant 5 | 33 | 9 | 8 | 29 | 17 | 4 | 31 | 10 | 9 |
| Participant 6 | 9 | 13 | 28 | 7 | 18 | 25 | 6 | 9 | 35 |
| Average | 25.17 | 13,67 | 11.67 | 19.67 | 21.83 | 8.5 | 23,33 | 14 | 12.67 |
